# Screening for medicine and food homologous ingredients targeting ALDH2 to alleviate acute ethanol-induced liver injury

**DOI:** 10.3389/fnut.2026.1913449

**Published:** 2026-09-29

**Authors:** Yuan Lu, Zibin Li, Pan Gao, Yushan Liu, Lin Han, Ji Bian, Min Wang

**Affiliations:** 1 College of Food Science and Engineering, Northwest A&F University, Yangling, Shaanxi, China; 2 Kolling Institute, Sydney Medical School, Royal North Shore Hospital, University of Sydney, Sydney, NSW, Australia

**Keywords:** alcoholic liver disease, ALDH2, hepatoprotection, medicine and food homologs, puerarin

## Abstract

**Background:**

Alcoholic liver disease (ALD) is a major global health burden with limited therapeutic options. Aldehyde dehydrogenase 2 (ALDH2) plays a critical role in alcohol metabolism, and ALDH2 deficiency exacerbates alcohol-induced hepatic injury.

**Aim:**

This study aimed to identify and validate natural ALDH2 activators from medicine and food homologous sources for alleviating acute ethanol-induced liver injury.

**Methods:**

A literature-based screening revealed 18 candidate compounds. *In vitro* enzyme kinetics, molecular docking, and isothermal titration calorimetry were employed to identify four potent ALDH2 activators from these 18 candidates. The hepatoprotective effects of the four candidates were validated *in vivo* using serum biochemical analyses, oxidative stress measurements, and histopathological examinations in the Gao-binge ALD mouse model.

**Results:**

Puerarin, sophoricoside, quercetin, and daidzin were preliminarily identified as mixed-type ALDH2 activators, which increased the acetaldehyde dehydrogenation rate catalyzed by ALDH2. These compounds significantly improved motor coordination, reduced blood ethanol and acetaldehyde levels, attenuated hepatic steatosis, normalized transaminase activities, restored antioxidant capacity, and ameliorated liver histopathological damage in the mouse model.

**Conclusion:**

Several natural ALDH2 activators from medicine and food homologous sources with potent hepatoprotective effects were identified, providing the scientific basis for developing healthy foods and dietary supplements to alleviate acute ethanol-induced liver injury.

## 1 Introduction

Alcoholic liver disease (ALD), one of the most common liver diseases globally, is caused by severe alcohol abuse. Approximately 23.6 million patients have compensated alcoholic cirrhosis, and 2.46 million patients have decompensated alcoholic cirrhosis worldwide (1). Recent improvements in living standards and lifestyle changes have led to an increased incidence of ALD. If the current drinking trends continue in China, more than 3.6 million people are predicted to develop alcoholic cirrhosis, leading to almost 2 million deaths due to alcoholic cirrhosis from 2022 to 2040 (2).

ALD progression correlates with the amount and duration of alcohol consumption and is affected by genetic and environmental factors. ALD initially presents as liver steatosis without clinical symptoms, which can develop into alcoholic hepatitis, alcoholic liver fibrosis and cirrhosis, and possibly liver failure and hepatocellular carcinoma (3). Unlike other liver diseases, ALD lacks specific diagnostic criteria. Clinical diagnosis mainly relies on a long-term heavy drinking history, examination results consistent with ALD, and exclusion of other liver diseases. The pathogenesis of ALD is not fully understood, and effective treatments are lacking. Thus, obtaining functional active ingredients from food to prevent and treat ALD may be an appropriate solution to treating ALD.

Aldehyde dehydrogenase 2 (ALDH2) is localized in the mitochondria, with the highest content in the liver. However, ALDH2 exhibits genetic polymorphism; individuals with low ALDH2 enzyme activity have a higher risk of developing acute ALD when consuming large amounts of alcohol (4). ALDH2 is also significantly associated with diseases or pathophysiological processes such as non-alcoholic fatty liver (5), liver cancer (6), and liver ischemia–reperfusion injury (7). Several mitochondrial signaling pathways affect the development of ALD by regulating ALDH2 activity. For example, nuclear factor E2-related factor 2 (Nrf2) knockdown significantly reduces ALDH2 expression in hepatocytes (8). The activated Nrf2/HO-1 and Nrf2/antioxidant response element signaling pathways can induce the expression of ALDH2 (9). ALDH2 can improve tumor-related diseases by mediating the AMPK and mTOR signaling pathways (10), and Alda-1 can protect the liver by inducing autophagy through the AKT/mTOR and AMPK signaling pathways (7). The current treatment for ALD relies on alcohol abstinence, nutritional support, and liver-protecting drugs, including silymarin and polyene phosphatidylcholine (11). However, poor patient compliance and drug side effects limit the long-term application of these therapies (12). Therefore, screening food sources for natural components that activate ALDH2 is an important strategy for developing alternative treatments for ALD.

Natural products have recently received extensive attention due to their multi-target and low-toxicity characteristics. In particular, medicine and food homologous components (such as *Pueraria lobata*, *Lycium barbarum*, and *Curcuma longa*) have traditional medicinal value and may be used as dietary supplements for the prevention and adjuvant treatment of ALD (13). Currently, the catalogs released by the National Health Commission of the People’s Republic of China and the State Administration for Market Regulation have included a total of 110 medicine and food homologous substances (14). The research and development of health foods using these substances as raw materials has become a development trend for people’s healthy living (15). Based on the screening of 90 papers published from 2016 to 2025, active ingredients targeting ALDH2 activation are under-investigated. Most studies focused on single components or mechanisms, without systematic screening for effects on ALDH2. Therefore, we screened for natural components that can activate ALDH2 and explored their regulatory mechanisms to identify new approaches to alleviate acute ethanol-induced liver injury. Natural bioactive compounds from medicine and food homologous and herbs were screened for activating effects on ALDH2 using pharmacological methods. *In vivo* animal experiments were conducted to verify the efficacy of candidates in relieving alcohol intoxication and protecting the liver. The results of this study provide new therapeutic strategies for acute ethanol-induced liver injury based on targeting ALDH2.

## 2 Materials and methods

### 2.1 Literature-based screening of medicine and food homologous active compounds

This study systematically retrieved four English databases, namely PubMed/MEDLINE, Scopus, Web of Science and Cochrane Library, as well as four Chinese databases, namely China National Knowledge Infrastructure (CNKI), Wanfang Database, China Science and Technology Journal Database, and Chinese Biomedical Literature CD-ROM Database. The retrieval time frame was from January 1, 2016 to May 15, 2025. The following keywords were adopted in the retrieval strategy: (“ALDH” or “ALDH2” and “activators” and “homology of medicine and food” or “medicine and food homologous” or “medicine and food homologous plant” or “medicine and food homologous materials”). Meanwhile, the references of the relevant studies or systematic reviews obtained from the retrieval were screened. Studies were excluded if they did not explicitly specify the specific substances exerting physiological activities, or if they only conducted network pharmacology analysis without performing cell experiments, animal experiments or human clinical trials. The molecules identified as activating ALDH2 are listed in Supplementary Table S1.

### 2.2 *In vitro* enzyme activity assay of ALDH2

To verify the effects of the identified candidates from the literature screening on ALDH, the modified Blair and Bodley method was used (16). In the presence of oxidized coenzyme I (NAD^+^), ALDH2 catalyzes the conversion of acetaldehyde and NAD^+^ into acetic acid and NADH, leading to an increase in absorbance at 340 nm. Thus, the activity of ALDH2 was calculated by measuring the change in absorbance at 340 nm. Briefly, in the assay tubes, 1.6 mL of 100 mmol/L sodium pyrophosphate buffer with a pH of 7.4, 1 mL of 3.6 mmol/L NAD^+^, 0.1 mL of 100 mmol/L acetaldehyde solution, and 0.1 mL of sample solution (dissolved in dimethyl sulfoxide) were added, respectively. Then, 0.1 mL of 18 mmol/L ALDH2 was added to the assay tubes. After shaking well, the OD_340nm_ value (A1) was immediately measured using a spectrophotometer. The tubes were placed in a water-bath at 37 °C, covered, and incubated for 5 min followed measuring the OD_340nm_ value (A2) immediately. According to the molar absorption coefficient of NADH at 340 nm, which is 6.22 × 10^−3^ L/μmol/cm, the enzyme activity units were calculated. The activity of ALDH2 was expressed as the number of nanomoles of NADH generated per minute. The formula for calculating ALDH2 activity is as follows: ALDH2 activity (U/mL) = (ΔA × *V*_total_)/(*ε* × *d* × *V*_sample_ × *T*); ΔA = A2–A1; *V*_total_: total reaction volume, 1 mL; ε: molar absorption coefficient of NADH, 6.22 × 10^−3^ L/μmol/cm; d: cuvette optical path length, 1 cm; *V*_sample_: added sample volume, 0.1 mL; T: reaction time, 5 min. The activity of ALDH2 was expressed as the number of nanomoles of NADH generated per minute. The formula for calculating ALDH2 activation rate is as follows: Activation rate (%) = (*A*_sample_ – *A*_control_)/*A*_control_ × 100%; *A*_sample_: ALDH2 activity with activator; *A*_control_: ALDH2 activity without activator. The test concentrations of each candidate compound were determined through preliminary experiments.

### 2.3 Enzymatic reaction kinetics of ALDH2

Kinetic experiments were conducted with different concentrations of active molecules, keeping the amount of ALDH2 constant (17). The different concentrations of active molecules were mixed with 3.6 mmol/L NAD^+^ solution, 100 mmol/L acetaldehyde solution, and 100 mmol/L sodium pyrophosphate buffer with a pH of 7.4, preheated at 37 °C for 5 min, and reacted with 18 mmol/L ALDH2 at 37 °C for 15 min. The absorbance was measured at 340 nm, and the activation type was preliminarily determined using the Lineweaver–Burk plot.

Isothermal titration calorimetry (ITC) measurements of enzyme kinetic reactions were conducted in the multi-injection titration mode. A solution of the substrate NAD^+^ (3.6 mmol/L) and acetaldehyde (100 mmol/L) was continuously injected into a solution of different concentrations of active molecules and ALDH2 (18 mmol/L). The substrate concentration was increased by continuous titration until the enzyme reached saturation and the maximum reaction velocity. By accurately measuring the change in heat over time and the compensation power and its integral, the reaction rate can be determined, and the activity of ALDH2 can be tracked in real time. The test concentrations of the four active molecules used in the enzyme kinetic reactions were determined through preliminary experiments; the experiments were based on the concentrations employed in the ALDH2 enzyme activity assay.

### 2.4 Molecular docking simulation

The interaction mode and mechanism for the reaction between the activators and ALDH2 were investigated using the Surflex-dock method. The three-dimensional crystal structure of ALDH2 was obtained from the PDB database (rcsb.org) with the accession number 3inl. After modifications, ALDH2 generated an active site in the form of a ligand, which was represented by the prototype molecule. New small-molecule compounds were prepared. The number of output conformations of the Surflex-dock ligand was set to 20, and the remaining parameters were set to default values. Ligand docking with ALDH2 was determined by referring to the original ligand structure. The total score was defined as the total scoring function for the output conformations, including the crash and polar scores. Output conformations with total scores higher than 6.000 were considered good output conformations.

### 2.5 Animal experiments

All animal procedures were approved by the Committee of Ethics Animal Experiments of Northwest A&F University (protocol code XN2024-0511; approval date: 11 May 2024) and were conducted in accordance with the institutional guidelines for the care and use of laboratory animals. An acute ethanol feeding model (Gao-binge model) (18) was established. C57BL/6 mice (SPF grade, female, 10–12 weeks of age; *n* = 110) were purchased from SpePharm (Beijing) Biotechnology Co., Ltd. (Beijing, China). The sample size was determined according to the number of experimental groups and the requirement to minimize experimental variability attributable to individual differences. Mice were fed a Lieber-DeCarli liquid diet for 5 days under conditions of 25 ± 1 °C, (60 ± 5)% relative humidity, and a 12/12 h light–dark cycle, with free access to diet, water, and movement, and were randomly divided into the following groups (*n* = 10 per group): a blank control group (C), an alcoholic liver injury model group (M), a positive control group (PC), high-dose puerarin, sophoricoside, quercetin, and daidzin treatment groups (PuH, SoH, QuH, and DaH, respectively), and low-dose of puerarin, sophoricoside, quercetin, and daidzin treatment groups (PuL, SoL, QuL, and DaL, respectively). Random numbers were generated using the standard = RAND () function in Microsoft Excel. Mice were fed a liquid diet containing 5% alcohol for 10 days except for group C. From the 13th day, mice in the PC group received Alda-1 (10 mg/kg body weight) by gavage, and mice in the high-dose and low-dose activator groups were given 10 mg/kg body weight or 5 mg/kg body weight, respectively, of activator 1 h before ethanol intragastric administration (19). Alda-1, namely N-(1,3-benzodioxol-5-ylmethyl)-2,6-dichlorobenzamide, is a small-molecule activator of ALDH2 identified through a high-throughput screening approach and has been shown to enhance ALDH2 activity via allosteric activation (20). Each mouse was managed by three independent researchers. The first researcher conducted the procedures according to the randomization schedule and was the only individual aware of the experimental group assignments. The second researcher was responsible for oral gavage administration. The third researcher, who was blinded to both group allocation and treatment conditions, measured and assessed the experimental outcomes. If oral gavage resulted in adverse effects, including feeding difficulties, decreased food intake, or body weight loss, the animals were monitored twice daily, and gavage administration was temporarily suspended as a supportive care measure. If these symptoms persisted despite supportive interventions, humane endpoint euthanasia was performed. On the 16th day, mice were given a single intragastric dose of high-concentration alcohol (5 g/kg body weight) except for group C. Mice were sacrificed by cervical dislocation method under anesthesia with 40 mg/kg body weight pentobarbital solution (intraperitoneally) on the following day. Blood and liver were collected.

### 2.6 Mouse behavior

#### 2.6.1 Rotating rod

The experiment was conducted with slight modifications based on the method of Tabuse et al. (21). Twelve minutes after intragastric administration of high-concentration alcohol, animals (n = 10 per group) were placed in a rotating rod fatigue tester (Shanghai Xinruan Information Technology Co., Ltd., Shanghai, China) with a rotation speed of 15 rotations/min (one mouse per chamber). Mice that could not stay on the stationary rod for more than 3 min were excluded. The time the mice first fell off the rotating rod during a 3-min period and the number of balance errors (i.e., the number of falls) were recorded.

#### 2.6.2 Climbing

Limb muscle strength and coordination of mice were evaluated using the climbing experiment. The method was adapted from the protocol described by Santos et al. (22). Immediately after alcohol administration, mice (*n* = 10 per group) were placed in the center of a horizontally suspended metal mesh, and the metal mesh was immediately turned over to make the mice hang upside down. Climbing movements were observed, and climbing times were recorded. The experiment ended when the mice fell three times, and the average of the three climbing times was calculated as the final experimental result.

#### 2.6.3 Pole-climbing

Limb coordination and bradykinesia were evaluated by pole-climbing. Faster pole climbing correlates with stronger limb grasping ability and better coordination. The protocol was modified from previous procedures (23). A wooden pole with a diameter of 15 mm and a length of 50 cm was used for the test. The surface of the pole was wrapped with gauze to increase friction and prevent the mice from slipping or sliding down the pole during climbing. Before the experiment, mice were trained to climb the pole until they could use their hind legs to hold the pole and crawl downwards. Mice that stopped while holding the pole or crawled in the reverse direction were excluded from the final results. After intragastric administration of high-concentration alcohol, mice (*n* = 10 per group) were placed head-down on the top of the pole. The total crawling time was recorded, and movement during crawling was observed. The experiment was repeated three times, and the average value was calculated as the crawling time of the mouse.

### 2.7 Detection of serum biochemical indicators in mice

Ethanol, acetaldehyde, total cholesterol (T-CHO), triglyceride (TG), alanine aminotransferase (ALT/GPT), and aspartate aminotransferase (AST/GOT) levels were detected in serum using kits (Nanjing Jiancheng Bioengineering Institute Co., Ltd., Nanjing, China) following the kits’ instructions.

### 2.8 Detection of oxidative stress indicators in mouse liver

ADH, ALDH, malondialdehyde (MDA), glutathione peroxidase (GSH-Px), and superoxide dismutase (SOD) in the liver were detected using kits (Nanjing Jiancheng Bioengineering Institute Co., Ltd., Nanjing, China) according to the kit instructions.

### 2.9 Staining of pathological sections of mouse livers

Part of the liver tissue was fixed in 4% paraformaldehyde fixative for 24 h. After dehydration, tissues were embedded in paraffin. The paraffin blocks were cut into 5-μm-thick sections using a microtome (MT1, Dakewe Biotech Co., Ltd., Shenzhen, China) and placed on adhesive slides. After incubating in a 60 °C incubator for 4 h, the sections were stained with hematoxylin and eosin (H&E) stains. After staining, an optical microscope (BX53MRF-S, Olympus, Japan) was used for observation and photography.

### 2.10 Statistical analysis

All experiments were conducted with three replicates, and the data are expressed as a mean ± standard deviation (*n* = 10 per group). One-way analysis of variance (ANOVA) was performed using SPSS 22.0 software, and *post hoc* LSD and Waller–Duncan tests were used for multiple comparisons. A *p*-value < 0.05 was considered significantly different.

## 3 Results

### 3.1 Discovery of targets activating ALDH2 from food and medicine homologous compounds

#### 3.1.1 Preliminary screening results

After extensive literature retrieval (more than 90 documents) from Web of Science and careful screening, we initially established a candidate library of 18 active compounds (Supplementary Table S1), including 12 flavonoids, 3 polyphenols, and 3 others (Supplementary Table S1). These active compounds are typically derived from *Pueraria lobata*, *Sophora japonica* flower, *Ginkgo biloba* L. leaf, Seed of *Hovenia dulcis* Thunb., *Glycine max* (L.) Merr., *Glycyrrhiza uralensis* Fisch, *Salvia miltiorrhiza* Bge., *Panax ginseng* C. A. Meyer, etc. To ensure accuracy and reliability, ALDH2 and the standards of the active compounds were purchased from Shanghai Yuanye Bio-Technology Co., Ltd., Shanghai, China. The *in vitro* activation of ALDH2 by the 18 candidate compounds was evaluated using spectrophotometry (Figure 1). We primarily screened candidate compounds based on the criteria that the activation rate was >40% at at least one experimental concentration and no negative activation rate was observed, through which five compounds, namely sophorabioside, puerarin, sophoricoside, quercetin and daidzin, were preliminarily identified. However, although both sophorabioside and sophoricoside are derived from *Sophora japonica* flower, the cost of sophorabioside is much higher than that of sophoricoside. Therefore, based on activation rates, sources and cost of the active compounds, puerarin, sophoricoside, quercetin, and daidzin were selected for further studies on alcoholic liver injury.

**Figure 1:**
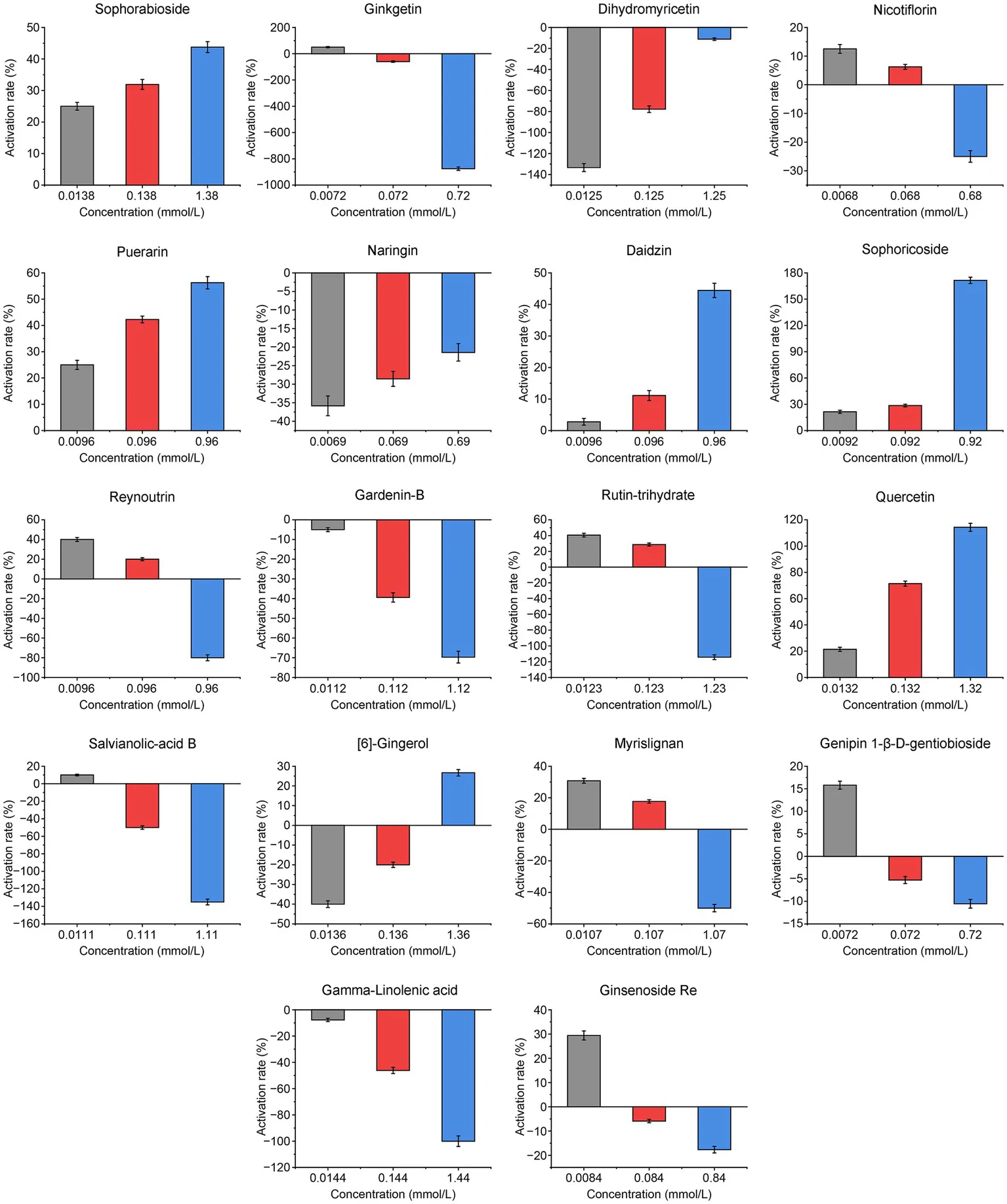
Activation rates of 18 active compounds on ALDH2 *in vitro*.

#### 3.1.2 *In vitro* enhancement of ALDH2 activity

To investigate the ALDH2 activation mechanisms of puerarin, quercetin, sophoricoside, and daidzin, nonlinear fitting curves and Lineweaver–Burk double-reciprocal curves of three different concentrations of the four activators were generated (Figures 2A–H). As shown in Table 1, the *Km* values gradually decreased with increasing concentrations of activators, but *Vmax* continuously increased. The three straight lines of the four activators intersected in the second quadrant. Moreover, the intercept on the vertical axis of the straight line decreased, and the slope gradually decreased as the activator concentration increased. These results indicate that the four activators might be mixed-type activators, including competitive and non-competitive activation. Thus, we preliminarily infer that the substrate NAD^+^ and the activators bind to ALDH2 simultaneously, and the activator competes with the substrate NAD^+^ for the binding site of the enzyme.

**Figure 2:**
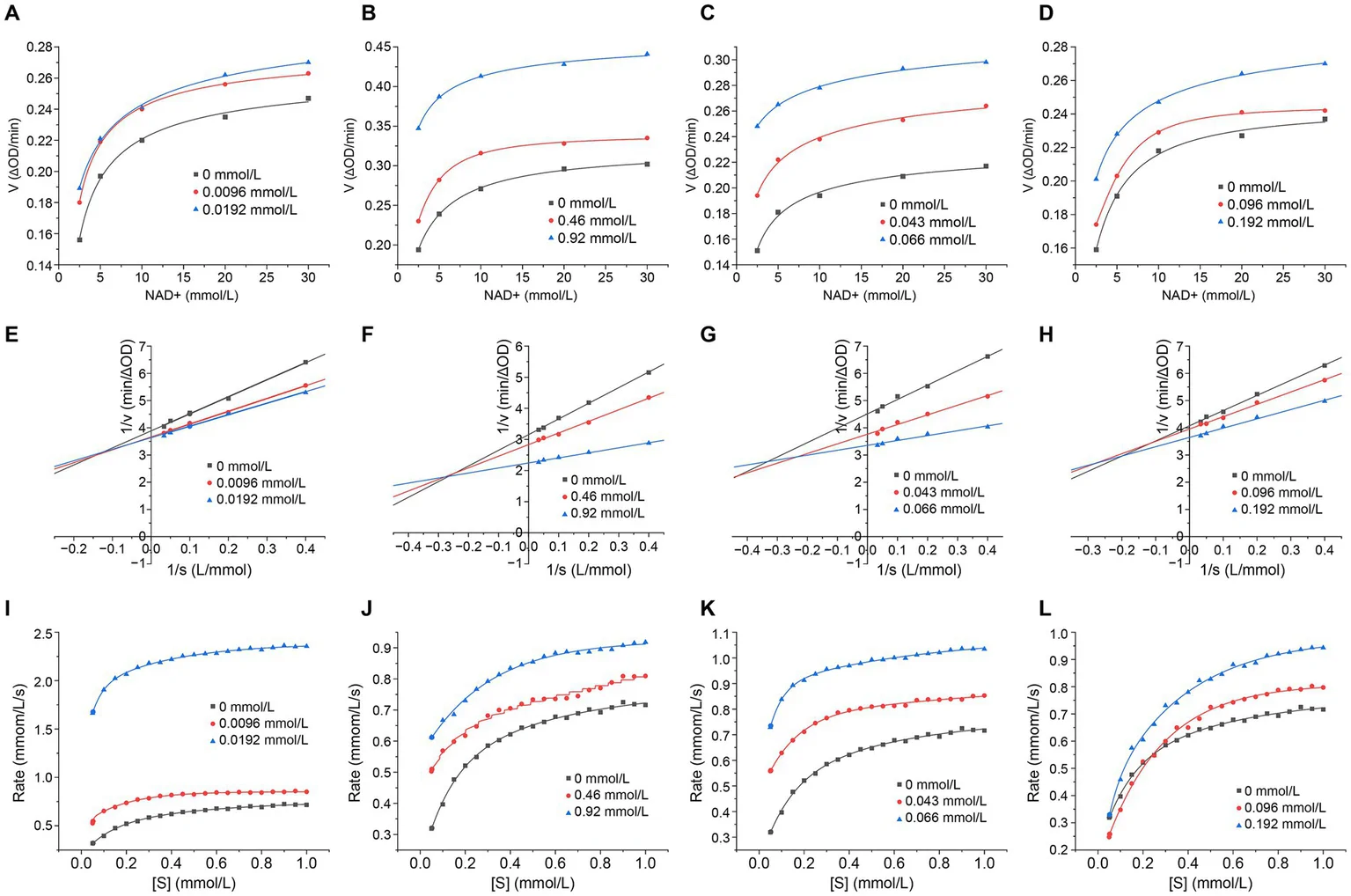
Nonlinear regression curves **(A–D)**, double-reciprocal activation plots **(E–H)**, and isothermal titration curves **(I–L)** for the effects of four active compounds on ALDH2. **(A,E,I)** Puerarin; **(B,F,J)** Sophoricoside; **(C,G,K)** Quercetin; and **(D,H,L)** Daidzin.

**Table 1:** Kinetic parameters associated with ALDH2 activation by four active compounds.

Compound	Concentration mmol/L	Lineweaver-Burk equation	*Km* mmol/L	*Vmax* OD_475 nm_/min
Puerarin	0	1/*V* = 6.260/[*S*] + 3.886	1.611	0.257
	0.0096	1/*V* = 4.709/[*S*] + 3.662	1.286	0.273
	0.0192	1/*V* = 4.231/[*S*] + 3.634	1.164	0.275
Sophoricoside	0	1/*V* = 5.036/[*S*] + 3.155	1.596	0.317
	0.46	1/*V* = 3.729/[*S*] + 2.834	1.316	0.353
	0.92	1/*V* = 1.619/[*S*] + 2.245	0.721	0.445
Quercetin	0	1/*V* = 5.282/[*S*] + 4.512	1.171	0.222
	0.043	1/*V* = 3.550/[*S*] + 3.764	0.943	0.266
	0.066	1/*V* = 1.793/[*S*] + 3.353	0.535	0.298
Daidzin	0	1/*V* = 5.579/[*S*] + 4.073	1.370	0.246
	0.096	1/*V* = 4.536/[*S*] + 3.954	1.147	0.253
	0.192	1/*V* = 3.415/[*S*] + 3.645	0.937	0.274

Figures 2I–L show the ITC curves for the acetaldehyde dehydrogenation reaction catalyzed by ALDH2 in phosphate buffer at pH 7.4 and 37 °C, in the presence or absence of different concentrations of activators. The reaction rate curves, as a function of substrate concentration, for different concentrations of activators were all above the blank curve (dilution buffer). Moreover, the reaction rates increased at higher activator concentrations, indicating that the rate of acetaldehyde dehydrogenation catalyzed by ALDH2 increased in the presence of the four activators. These results confirmed that puerarin, sophoricoside, quercetin, and daidzin activate ALDH2.

#### 3.1.3 Molecular docking simulation

Precise molecular docking simulations were conducted for the four active compounds and ALDH2, and key parameters, including energy changes, binding sites, and interaction modes during docking, were calculated. Comprehensive scores for the binding of each active compound to ALDH2 were determined. Figure 3 shows the molecular docking diagrams of the four active compounds with ALDH2. The total scores of puerarin, sophoricoside, quercetin, and daidzin were 7.37, 6.60, 6.22, and 7.11, respectively. The binding free energy of puerarin, sophoricoside, quercetin, and daidzin were −9.72, −9.47, −9.40, and −9.64 kcal/mol, respectively. As shown in Figure 3A, puerarin interacts with ALDH2 via hydrogen bonds involving Gly474, Val488, Tyr139, Thr486, Gln483, and Gln254; as shown in Figure 3B, sophoricoside interacts with ALDH2 via hydrogen bonds involving Cys301, Cys203, Phe296, Asp457, Val458, and Gly460. Furthermore, quercetin interacts with ALDH2 through hydrogen bond interactions involving Thr185, Ala184, Asn499, Lys498, Asp147, and Ile146 (Figure 3C); daidzin interacts with ALDH2 through hydrogen bond interactions involving Cys301, Cys303, Asn169, Phe296, Phe459, and Val458 (Figure 3D). In summary, puerarin, sophoricoside, quercetin, and daidzin interact with ALDH2 through hydrogen bonds, binding to the active site of the target protein and exhibiting good binding activity with the target protein.

**Figure 3:**
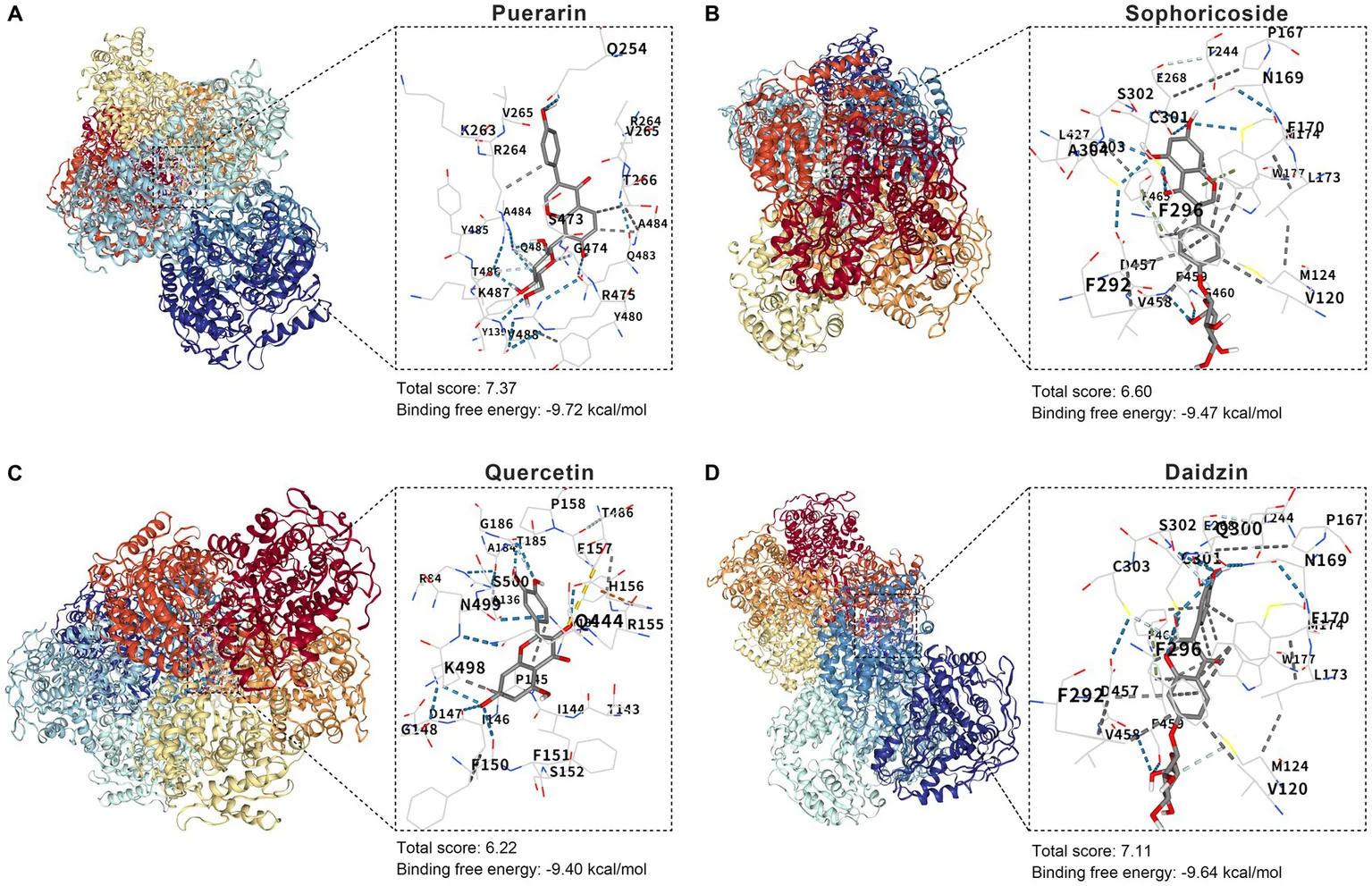
Molecular docking diagrams of the four active compounds with ALDH2. **(A)** Puerarin; **(B)** Sophoricoside; **(C)** Quercetin; **(D)** Daidzin.

### 3.2 Activators attenuated the impaired motor coordination in ethanol-intoxicated mice

The animal experiment procedure is shown in Figure 4, and the mouse behavioral tests were conducted after intragastric administration of 95% ethanol (5 g/kg BW) on day 17. The mice in group C exhibited a good mental state, relatively agile responses, and no signs of drunkenness. After treatment with high-concentration alcohol by gavage, the mice in group M exhibited abnormal behaviors, including poor mental state, dragging of the hindquarters, and ataxia. These behaviors were significantly improved after drug administration. As shown in Figures 5A,B, the retention time on the rotating rod was significantly shortened (*p* < 0.001) and the number of falls from the rotating rod was significantly increased (*p* < 0.001) in group M compared with group C. The retention times on the rotating rod were significantly increased (*p* < 0.05), and the number of falls from the rotating rod was significantly reduced (*p* < 0.001) in the PC group and the high- and low-dose activator groups compared with group M. These results suggest that puerarin, sophoricoside, quercetin, and daidzin improve the balance disorder in mice caused by ethanol.

**Figure 4:**
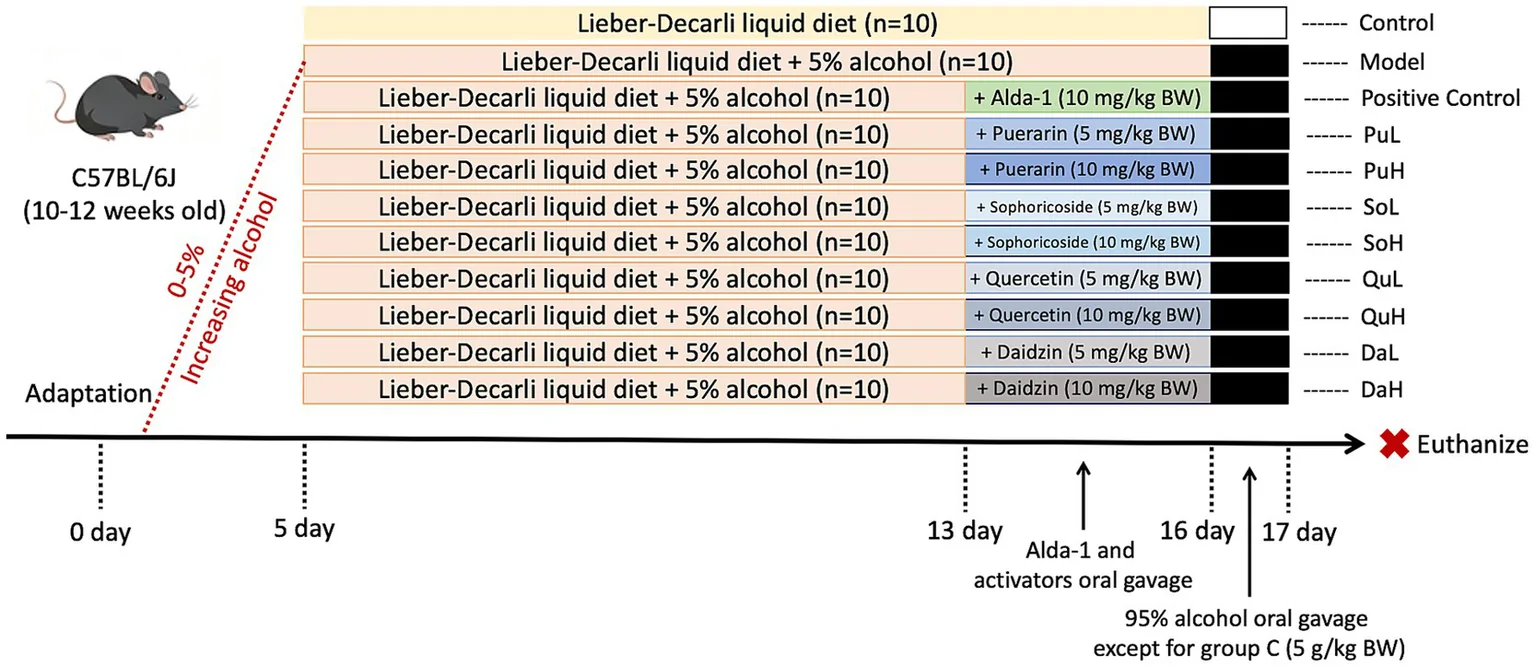
The animal experiment procedure.

**Figure 5:**
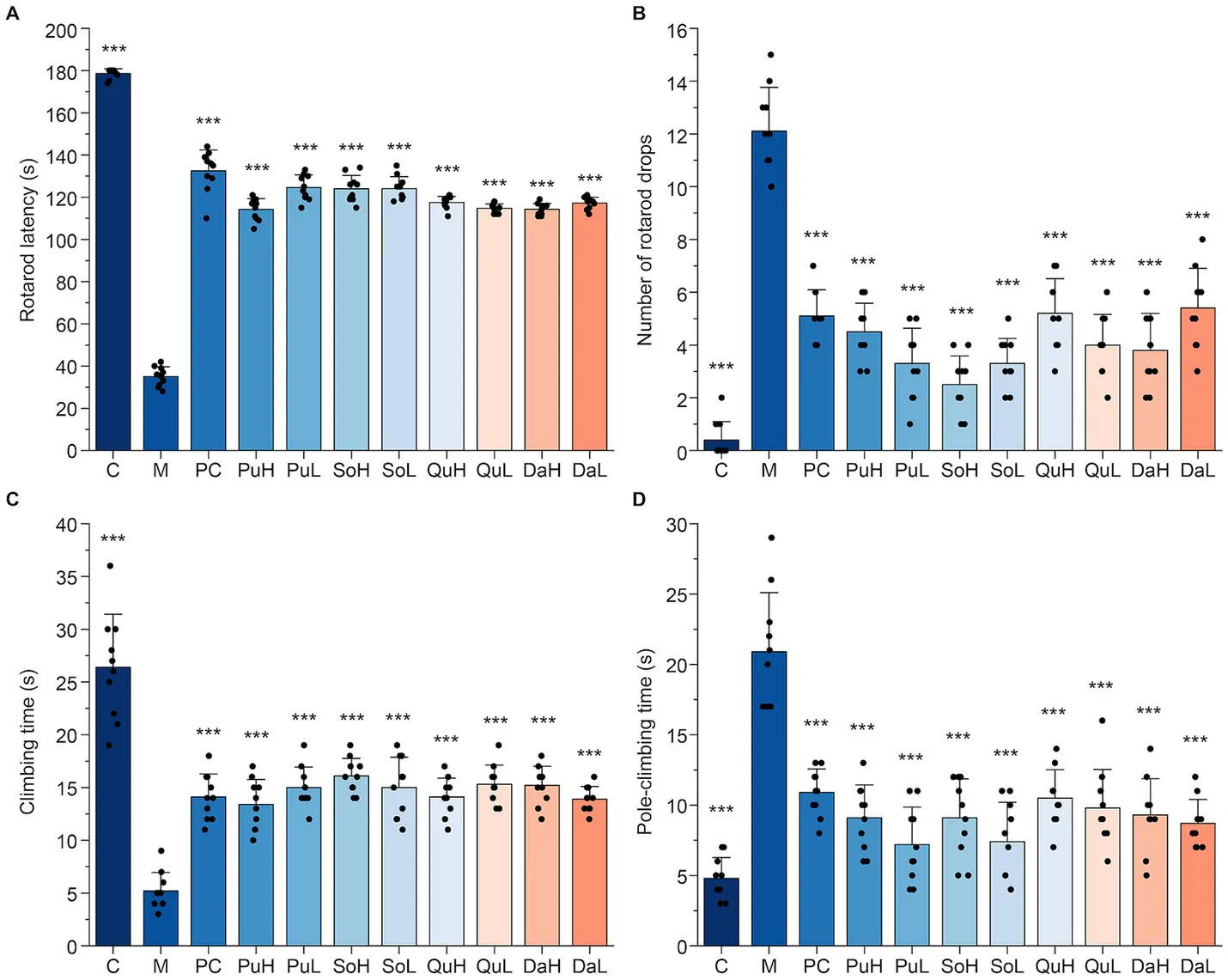
Effects of activators on motor coordination function in mice after drunkenness. **(A)** Rotarod latency; **(B)** Number of rotarod drops; **(C)** Climbing time; **(D)** Pole-climbing time. C, control group; M, alcoholic liver injury model group; PC, positive control group (Alda-1 treatment group); PuH, SoH, QuH, and DaH, high-dose of puerarin, sophoricoside, quercetin, and daidzin treatment groups; PuL, SoL, QuL, and DaL, low-dose of puerarin, sophoricoside, quercetin, and daidzin treatment groups. Data are presented as the mean ± SD (*n* = 10), and multiple comparisons between M group and other groups were analyzed using ANOVA followed by *post hoc* LSD and Waller-Duncan tests. ***p < 0.001 compared with group M.

As shown in Figure 5C, the climbing times were shorter for mice in group M compared with the times for mice in group C (*p* < 0.001). The climbing times for mice in the PC and high- and low-dose groups of the four activators were significantly increased (*p* < 0.001) compared with the times for mice in group M; no significant differences were detected between the activator-treated and PC groups. These results indicate that puerarin, sophoricoside, quercetin, and daidzin improve motor coordination and muscle strength in ethanol-intoxicated mice.

Pole-climbing times in group M significantly increased compared with group C (*p* < 0.001). The pole-climbing times of mice in the PC and the high- and low-dose groups of the four activators all decreased significantly compared with group M (*p* < 0.001); the PuL and SoL activator treatment groups were superior to the PC group (Figure 5D). These results suggest that puerarin, sophoricoside, quercetin, and daidzin improved bradykinesia and limb coordination and balance.

### 3.3 Activators reduced ethanol, acetaldehyde, triglycerides, and total cholesterol levels and transaminase activities in the serum

Most ethanol is absorbed by the gastrointestinal tract and reaches the liver for metabolism via the circulation (24). The blood ethanol concentrations in the different treatment groups are shown in Table 2. The blood ethanol concentrations of mice in group M were significantly higher than the concentrations in group C (*p* < 0.001). The circulating ethanol concentrations of mice in the PC group and the high- and low-dose activator groups were significantly reduced compared with the concentrations in group M (*p* < 0.01, *p* < 0.05). These results demonstrate that the four activators can markedly reduce the ethanol content in the blood of mice.

**Table 2:** Effects of activators on serum biochemical indices in mice.

Group	Alcohol mg/100 mL	Acetaldehyde μg/mL	TG mmol/L	T-CHO mmol/L	ALT U/L	AST U/L
C	2.72 ± 0.65***	0.66 ± 0.08***	1.69 ± 0.04***	1.73 ± 0.03***	49.37 ± 5.03***	162.83 ± 14.19***
M	465.21 ± 34.77	57.02 ± 3.96	3.70 ± 0.26	3.19 ± 0.06	124.88 ± 13.91	303.70 ± 24.36
PC	319.82 ± 16.04**	26.78 ± 1.58***	2.28 ± 0.07**	2.13 ± 0.07***	77.62 ± 5.74**	198.44 ± 14.23**
PuH	339.56 ± 9.51**	32.38 ± 2.62***	2.36 ± 0.11**	2.19 ± 0.10***	71.70 ± 9.37**	243.14 ± 24.74*
PuL	393.21 ± 16.40*	39.25 ± 3.17**	2.13 ± 0.15***	2.04 ± 0.14***	68.92 ± 3.53**	234.17 ± 9.36**
SoH	396.32 ± 12.36*	40.15 ± 2.85**	1.81 ± 0.23***	2.03 ± 0.12***	69.21 ± 5.44**	205.50 ± 19.86**
SoL	348.15 ± 10.54**	34.69 ± 2.58**	1.74 ± 0.09***	2.01 ± 0.06***	68.21 ± 4.85**	178.78 ± 11.24**
QuH	388.63 ± 16.35*	40.56 ± 3.01**	2.14 ± 0.18**	2.07 ± 0.03***	77.05 ± 9.01**	257.67 ± 1.92*
QuL	357.89 ± 13.65**	35.25 ± 2.74**	1.97 ± 0.07***	2.07 ± 0.17***	73.27 ± 9.19**	238.91 ± 31.37*
DaH	401.23 ± 15.48*	44.16 ± 3.25*	2.29 ± 0.15**	2.37 ± 0.04***	84.03 ± 5.20**	207.59 ± 11.55**
DaL	371.58 ± 10.69*	38.27 ± 2.78**	2.38 ± 0.13**	2.28 ± 0.08***	76.86 ± 5.47**	215.61 ± 15.03**

Data are presented as the mean ± SD (*n* = 10), and multiple comparisons between M group and other groups were analyzed using ANOVA followed by *post hoc* LSD and Waller–Duncan tests. Compared with the M group, *p < 0.05, **p < 0.01, ***p < 0.001.

Ethanol is oxidized by liver alcohol dehydrogenase to toxic acetaldehyde, which is oxidized to non-toxic acetic acid by aldehyde dehydrogenase and further oxidized to water and carbon dioxide before being excreted from the body (25). The acetaldehyde concentrations in the blood of mice in the PC group and high- and low-dose activator groups were significantly reduced compared with group M (*p* < 0.05). Although acetaldehyde concentrations in the activator-treated groups were not reversed compared to the positive control group, the decreasing trend indicates that activators can significantly reduce circulating acetaldehyde content in mice, possibly by increasing aldehyde dehydrogenase activity and accelerating acetaldehyde decomposition (26). As shown in Table 2, the serum TG content of mice in group M was significantly higher than the TG content in group C (*p* < 0.001). The TG content was significantly reduced by treatment with Alda-1 and the activators (*p* < 0.05), but was not restored to normal levels compared to the C group. No significant differences in TG-lowering activity were detected between activators and the PC group. The serum T-CHO content was significantly higher in the M group compared with the C group (*p* < 0.001) (Table 2) and was attenuated by activator treatment (*p* < 0.05). This result indicates that activators can reduce cholesterol to levels close to those of the PC group. Similarly, serum ALT and AST levels were lower in the four activator-treated groups compared with the M group (*p* < 0.05), indicating that oral administration of the activators elicited hepatic protective activity against acute alcohol injury.

### 3.4 Activators improved liver indices in mice by enhancing catalytic activities of alcohol-metabolizing enzymes and reducing oxidative stress responses

The liver index is closely related to alcohol-induced liver injury, reflecting the degree of liver injury (27). As shown in Table 3, the liver index of mice in the M group increased dramatically compared with that of mice in the C group (*p* < 0.001), indicating that the alcoholic liver injury model was successfully established. Intragastric administration of the positive drug (Alda-1) and different doses of puerarin, sophoricoside, quercetin significantly reduced the liver index compared with the M group (*p* < 0.05). However, although the liver index in the high- and low-dose daidzin groups decreased, there was no significant difference compared with the M group. Moreover, the liver index in the high- and low-dose daidzin groups was higher than that in the PC group, while all other activator groups were lower than the PC group. These results suggest that the activators can reduce the liver index and alleviate acute ethanol-induced liver injury in mice.

**Table 3:** Effects of activators on liver index, ADH, ALDH, oxidative stress levels, lipid, and steatosis of liver in mice.

Group	Liver index %	ADH U/mg prot	ALDH U/mg prot	MDA nmol/mg prot	T-SOD U/mgprot	GSH-Px U/mg prot	TG mg/g tissue	T-CHO mg/g tissue	Steatosis (%/200 μm)
C	3.62 ± 0.13***	264.14 ± 33.12***	551.55 ± 26.40*	26.57 ± 2.43***	802.59 ± 18.13***	2776.10 ± 27.92**	23.05 ± 0.24***	7.14 ± 0.14*	0.00 ± 0.00***
M	3.96 ± 0.18	577.38 ± 36.00	639.82 ± 33.92	51.93 ± 2.22	689.93 ± 12.88	2574.14 ± 67.43	44.35 ± 0.17	8.15 ± 0.15	14.59 ± 0.18
PC	3.75 ± 0.16*	697.03 ± 73.82*	859.47 ± 28.56***	38.55 ± 0.44***	733.26 ± 12.63*	2746.03 ± 10.43*	32.09 ± 0.21**	8.03 ± 0.21	0.34 ± 0.05***
PuH	3.71 ± 0.17*	589.82 ± 17.97	853.88 ± 20.61***	47.31 ± 4.10	739.36 ± 20.54*	2774.13 ± 98.57*	34.21 ± 0.20**	8.12 ± 0.11	0.31 ± 0.08***
PuL	3.63 ± 0.18**	678.71 ± 31.49*	656.47 ± 28.32	44.72 ± 3.04*	790.26 ± 22.60**	2764.81 ± 40.56*	33.92 ± 0.21**	7.47 ± 0.16*	0.35 ± 0.09***
SoH	3.62 ± 0.17***	704.75 ± 59.89*	693.92 ± 37.37	44.54 ± 3.37*	770.87 ± 22.15**	2819.85 ± 75.46*	33.15 ± 0.18**	7.72 ± 0.1	0.25 ± 0.06***
SoL	3.66 ± 0.16**	765.61 ± 20.59**	861.21 ± 62.88***	35.02 ± 2.11***	855.81 ± 28.47***	2963.28 ± 71.50**	28.22 ± 0.20**	7.7 ± 0.19	0.24 ± 0.10***
QuH	3.68 ± 0.17**	608.70 ± 30.40	640.73 ± 75.32	47.21 ± 5.15	782.93 ± 16.16**	2529.05 ± 33.98	34.43 ± 0.26**	7.53 ± 0.09*	0.30 ± 0.07***
QuL	3.66 ± 0.26**	655.04 ± 43.77	720.81 ± 53.45*	43.25 ± 0.26*	744.14 ± 30.08*	2752.88 ± 58.66*	29.12 ± 0.26**	7.36 ± 0.16*	0.27 ± 0.08***
DaH	3.82 ± 0.20	634.90 ± 15.52	710.91 ± 20.23*	47.21 ± 6.70	759.46 ± 36.13*	2675.19 ± 74.93	34.48 ± 0.22**	7.28 ± 0.19*	0.32 ± 0.07***
DaL	3.86 ± 0.15	580.11 ± 19.47	745.18 ± 17.41**	48.75 ± 2.08	750.80 ± 10.60**	2765.15 ± 87.74*	29.38 ± 0.19**	7.27 ± 0.19*	0.23 ± 0.06***

Data are presented as the mean ± SD (*n* = 10), and multiple comparisons between M group and other groups were analyzed using ANOVA followed by *post hoc* LSD and Waller–Duncan tests. Compared with group M, *p < 0.05, **p < 0.01, ***p < 0.001.

The ADH and ALDH activities in the livers of mice in the M group increased significantly compared with the activities in the C group (*p* < 0.001, *p* < 0.05) (Table 3). Treatment with Alda-1 and puerarin, sophoricoside, quercetin, and daidzin increased ADH activities to varying degrees. However, compared with the M group, only the PC, PuL, SoH, and SoL groups showed significant differences (*p* < 0.05, *p* < 0.01), while the others did not. Similarly, the ALDH activity in all treatment groups was higher than that in the M group, but there were no significant differences between M group and PuL, SoH, and QuH groups. High-dose puerarin stimulated ALDH more than the low-dose group (*p* < 0.05), and ALDH activity in the low-dose quercetin, sophoricoside, and daidzin groups was significantly higher than the activity in the high-dose group (*p* < 0.05). These results indicate that the four activators increase ADH and ALDH activities in the mouse liver, and their efficacy differed to some extent depending on the dosage. Among all activator treatments, only low-dose sophoricoside exerted an enhancing effect on the activities of ADH and ALDH that reached or exceeded that of the positive drug (Alda-1).

Alcohol exposure significantly increased MDA levels in the mouse liver (*p* < 0.05), exacerbating lipid peroxidation injury. High- and low-dose activators and Alda-1 treatment reduced MDA levels, but statistically significant reductions were observed only in the PC, PuL, SoH, SoL, and QuL groups (*p* < 0.05, *p* < 0.001). The activator effects were weaker than the effects of Alda-1 except for SoL group, and no activator dose-dependence was detected (Table 3). Ethanol also significantly inhibited GSH-Px and SOD activities in the liver (*p* < 0.05). GSH-Px and SOD activities completely recovered to normal levels after treatment with the four activators. In the treatments with puerarin and sophoricoside, low-dose treatment exhibited a superior effect on enhancing SOD activity compared to high-dose treatment; conversely, in the treatments with quercetin and daidzin, high-dose treatment showed a better effect on elevating SOD activity than low-dose treatment. Regarding the enhancement of GSH-Px activity, with the exception of puerarin treatment, low-dose treatments of sophoricoside, quercetin, and daidzin all outperformed their corresponding high-dose treatments. Overall, the antioxidant stress capacity of low-dose sophoricoside treatment exceeded that of the positive drug (Alda-1).

### 3.5 Activators improved pathological changes in mouse livers

The degree and characteristics of tissue lesions and injuries were determined by histopathological examination of H&E-stained liver sections. As shown in Figure 6, the hepatocytes in the control group were arranged neatly with intact morphological structures, normal nuclei, and no fatty degeneration. The morphological structure of liver cells was incomplete in the M group; the arrangement and the boundaries between cells were disordered and blurred, with extensive vacuolar degeneration, which was also manifested as a significant increase in liver TG content (*p* < 0.01) (Table 3). The PC group and the high- and low-dose activator groups exhibited reduced TG content and steatosis significantly (*p* < 0.01, *p* < 0.001) compared with the M group (Table 3). However, no lobular inflammation or ballooning degeneration was observed in the livers of any of the mice, which might be attributed to the short-term alcohol feeding model. These results indicate that puerarin, sophoricoside, quercetin, and daidzin improve acute ethanol-induced liver injury.

**Figure 6:**
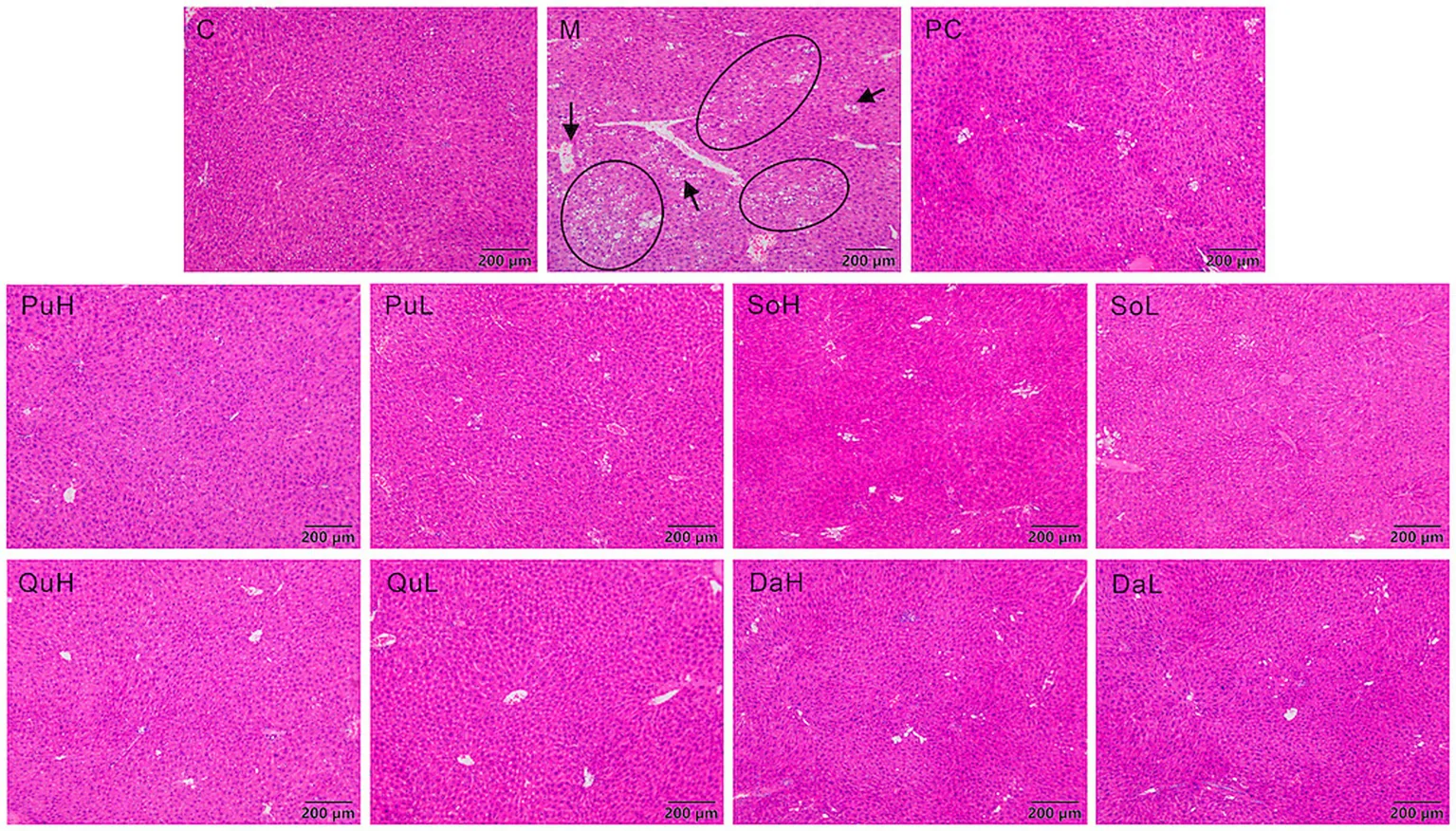
Hematoxylin and eosin staining images of the livers of mice in each treatment group. C, control group; M, alcoholic liver injury model group; PC, positive control group (Alda-1 treatment group); PuH, SoH, QuH, and DaH, high-dose of puerarin, sophoricoside, quercetin, and daidzin treatment groups; PuL, SoL, QuL, and DaL, low-dose of puerarin, sophoricoside, quercetin, and daidzin treatment groups.

## 4 Discussion

The goal of this study was to explore medicine and food homologous compounds that alleviate acute ethanol-induced liver injury. To achieve this goal, we systematically identified active compounds closely related to ALDH2 via a meticulous literature review. Flavonoids exhibit remarkable antioxidant and anti-inflammatory activities (28, 29), polyphenols scavenge free radicals (30), and alkaloids have unique pharmacological effects (31). We initially established a candidate library of 18 active compounds from a wide range of sources, including various plants and foods. Measuring the *in vitro* activation rate of ALDH2 and combining activation kinetics and ITC analysis preliminarily demonstrated that puerarin, quercetin, sophoricoside, and daidzin might be potent mixed-type activators of ALDH2, a key enzyme in the alcohol detoxification process. Molecular docking analysis showed that puerarin has the strongest binding capacity to the active site of ALDH2 among the four compounds. *In vivo* animal experiments showed that the four natural activators alleviated alcohol-induced liver injury in mice via multi-target regulation of ethanol metabolism, lipid homeostasis, hepatocyte integrity, alcohol-metabolizing enzymes, and antioxidant defense systems.

In the past 30 years, pre-screening compound libraries through computational methods, such as high-throughput virtual screening, has gradually been incorporated into drug discovery strategies (32, 33). In this study, *in vitro* enzyme activity assays, enzyme kinetics, and thermodynamic analyses were combined with molecular docking, a virtual screening technology, to identify the strongest four food and medicine homologous compounds (puerarin, quercetin, sophoricoside, and daidzin) that activate ALDH2. *In vivo* experiments were conducted to verify the activities of the four compounds. Zhang et al. (34) integrated transcriptome sequencing, network pharmacology, and molecular docking technology to demonstrate that puerarin, daidzein, schisandrol A, and schisandrin A from *Pueraria lobata* and *Schisandra chinensis* improve ALD by modulating the PI3K/AKT pathway. Dong et al. (35) reported the identification of sesquiterpenes from *Curcuma longa* and explored their potential against ALD using feature-based molecular networking, network pharmacology, molecular docking, and cell-based validation. Chen et al. (36) predicted the pharmacodynamic components and molecular mechanisms of Ge-Zhi-Jie-Jiu decoction (JJY) against ALD using network pharmacology and molecular docking methods. The results showed that the main components of JJY are organic acids, flavonoids, and isoflavonoids, and puerarin, daidzein, glycitin, formononetin, quercetin, and irigenin reflect the characteristic components of JJY. Artificial intelligence can significantly improve the precision and accuracy (37) of virtual screening to rapidly screen and optimize candidate drugs.

Medicine and food homologous compounds offer distinct advantages for alleviating alcoholic liver injury. Unlike synthetic pharmaceuticals, the dietary safety of these natural products has been established and can be readily incorporated into foods or nutraceutical formulations (38). Puerarin, derived from *Pueraria lobata* (kudzu), is traditionally used in East Asian medicine for alcohol intoxication relief (39). Quercetin, which is abundant in onions and apples, exhibits broad-spectrum anti-inflammatory and antioxidant properties (40). Daidzin, an isoflavone from soybeans (41), and sophoricoside from *Sophora japonica* (42) are additional examples of dietary phytochemicals with pharmacological potential. The convergence of traditional medicinal knowledge with modern molecular target validation provides a robust framework for developing evidence-based functional foods targeting ALD (38). In the Gao-binge mouse model, the *in vivo* efficacy of these natural activators was demonstrated through multiple biochemical assessments. These improvements in biochemical indicators correlated with reduced blood ethanol and acetaldehyde concentrations, suggesting that enhanced ALDH2 activity facilitates rapid alcohol clearance and reduces systemic acetaldehyde exposure.

Alda-1 is an agonist of ALDH2; its primary function is to activate mitochondrial ALDH2, including both wild-type and mutant forms, thereby enhancing its capacity to metabolize toxic aldehydes and reducing oxidative stress–induced damage (20). This mechanism also underlies its widespread application in studies of ALDH2-related diseases. For example, in a liver ischemia–reperfusion (I/R) model, Alda-1 pretreatment reduced transaminase levels, attenuated histopathological liver injury, suppressed the accumulation of reactive oxygen species (ROS), pro-inflammatory mediators, and toxic aldehydes (4-HNE and MDA), and decreased apoptosis, without altering ALDH2 expression levels but instead enhancing its enzymatic activity (43). Similarly, in a rat model of post-myocardial infarction cardiomyopathy, Alda-1 reduced aldehyde overload, improved mitochondrial function, and enhanced ventricular performance (44). Notably, the hepatoprotective effects of four compounds in the present study were comparable to Alda-1, a first-generation synthetic ALDH2 activator; however, compounds derived from natural food sources have superior safety profiles.

The hepatoprotective mechanisms extend beyond the enhancement of alcohol metabolism. Our results demonstrated that these natural compounds significantly attenuated alcohol-induced hepatic steatosis, as evidenced by reduced serum triglycerides and total cholesterol levels, and normalized liver enzyme profiles (ALT and AST). The histopathological improvements, including reduced vacuolar degeneration and restored hepatocyte architecture, confirmed the protective effects of the activators against alcohol-induced cellular damage. These findings align with recent studies highlighting the multi-targeted protective effects of flavonoids against ALD (39). Oxidative stress is a critical pathogenic mechanism contributing to ALD progression (45). Chronic alcohol exposure depletes hepatic antioxidant reserves and exacerbates lipid peroxidation, culminating in mitochondrial dysfunction and hepatocyte apoptosis (46). Our study demonstrated that all four natural activators restored hepatic antioxidant capacity, as evidenced by normalized GSH-Px and SOD activities and reduced MDA levels. These antioxidant effects likely synergize with ALDH2 activation to provide comprehensive hepatic protection. ALDH2 activation can attenuate oxidative stress by upregulating the Nrf2 pathway, suggesting crosstalk between ALDH2 enhancement and cellular antioxidant defenses (47). Yang et al. (48) reported that cucurbitacin B ameliorated acetaminophen-induced mitochondrial oxidative stress via the Nrf2 pathway. Chen et al. (49) also reported that α-asarone enhanced the antioxidant capacity of mice with valproic acid-induced liver injury via the Nrf2 pathway. However, these studies on drug-induced liver injury are not related to the specific mechanisms of alcoholic liver disease and ALDH2 specificity.

Several limitations to this study warrant consideration. First, the activation types of the four compounds against ALDH2 were only preliminarily inferred from double-reciprocal curves. Future studies still require more data such as quantitative kinetic fitting and structural simulation to fully confirm the competitive binding relationship between the compounds and NAD^+^. Second, the acute Gao-binge model is an excellent model for studying early ALD pathogenesis, but it does not fully recapitulate chronic disease progression, involving fibrosis and cirrhosis (50, 51). The current study demonstrates limited protection of liver structure; however, these findings suggest that the four natural activators may have potential as metabolic modulators. In future studies, the activator compounds should be evaluated in chronic-plus-binge or Lieber-DeCarli liquid diet models to assess long-term efficacy and anti-fibrotic capacity. Third, the acute Gao-binge model only caused mild hepatic steatosis. Oil Red O staining was not performed in this study and will be supplemented in subsequent long-term animal experiments. Fourth, intestinal microbiota detection was not performed in this study, which constitutes a research limitation of the absence of mechanisms related to the gut-liver axis. The precise molecular mechanisms underlying ALDH2 activation by these compounds should be elucidated through site-directed mutagenesis and multi-omics studies, such as gut microbiome and metabolomics (52).

Furthermore, the cellular and in-vivo pharmacokinetic characteristics of these four candidate compounds remain to be clarified. Although all four compounds showed direct ALDH2-activating effects in the present in-vitro enzyme assays, their in-vivo biological effects are also influenced by absorption, tissue distribution, cellular uptake, and hepatic exposure. Previous pharmacokinetic and tissue-distribution studies have demonstrated that intact puerarin and daidzin can be detected in liver tissue following oral administration, while quercetin and its derivatives have also been detected in hepatic tissue, supporting the possibility of hepatic exposure to these compounds (53–56). In addition, flavonoids undergo extensive biotransformation, including phase II conjugation in tissues such as the liver and intestine (57). However, the present study did not directly determine the plasma or hepatic concentrations of puerarin, sophoricoside, quercetin, or daidzin, nor did it evaluate their intracellular concentrations in hepatocytes. Therefore, the relationship between systemic or hepatic exposure and the observed in-vivo ALDH2-related effects remains to be established. In addition, intestinal microbiota were not characterized in the present study, and their potential contribution to the absorption and biotransformation of these compounds and to the gut–liver axis remains unclear. Future studies should integrate plasma and tissue pharmacokinetic analyses, hepatocyte uptake experiments, metabolite profiling, and gut microbiota characterization to further clarify the in-vivo exposure characteristics and pharmacological mechanisms of these natural ALDH2 activators.

The clinical implications of our findings are substantial. Given the high prevalence of ALDH2*2 polymorphism in East Asian populations (approximately 30–50%), identifying safe and effective ALDH2 activators from dietary sources offers a personalized prevention strategy for ALD-susceptible individuals (58). Furthermore, the multi-component nature of traditional herbal formulations suggests potential synergistic interactions between the identified compounds and other bioactive constituents, warranting investigation into optimized combination therapies (59). In conclusion, four natural ALDH2 activators from medicine and food homologous sources with potent protective effects against acute alcohol-induced liver injury were identified and validated. These findings provide the scientific foundation for developing foods and dietary supplements for alleviating acute ethanol-induced liver injury.

## 5 Conclusion

A systematic screening platform integrating literature-based screening, *in vitro* enzyme kinetics, molecular docking simulation, and *in vivo* pharmacological validation was successfully established to identify natural ALDH2 activators from medicine and food homologous sources. After comprehensive screening of 18 candidate compounds, four potent ALDH2 activators, including puerarin, sophoricoside, quercetin, and daidzin, with significant hepatoprotective effects against acute alcohol-induced liver injury were identified. Our preliminary mechanistic investigations revealed that these four compounds might function as mixed-type ALDH2 activators, simultaneously enhancing substrate affinity and catalytic efficiency to accelerate acetaldehyde detoxification. Molecular docking analyses confirmed the stable binding of these activators to the ALDH2 active site; puerarin exhibited the highest binding affinity. *In vivo* experiments using the Gao-binge model demonstrated that these natural activators significantly improved alcohol-induced motor coordination deficits, reduced blood ethanol and acetaldehyde concentrations, attenuated hepatic steatosis, and normalized liver enzyme profiles. Furthermore, these compounds effectively restored hepatic antioxidant capacity by upregulating GSH-Px and SOD activities and reducing lipid peroxidation, ultimately ameliorating histopathological damage.

The identification of safe and effective ALDH2 activators from medicine and food homologous sources offers promising translational potential. These findings provide the scientific foundation for developing healthy foods and dietary supplements for alleviating acute ethanol-induced liver injury based on the ALDH2 molecular target. Future research should focus on chronic ALD models, long-term safety evaluations, and clinical trials to fully realize the therapeutic potential of these natural ALDH2 activators in personalized ALD intervention strategies.

## Data Availability

The original contributions presented in the study are included in the article/Supplementary material, further inquiries can be directed to the corresponding authors.
