# Supplementary material for: Screening for medicine and food homologous ingredients targeting ALDH2 to alleviate acute ethanol-induced liver injury
Source: Front Nutr. 2026 Sep 29;13:1913449. doi: 10.3389/fnut.2026.1913449 (PMC13658622; doi:10.3389/fnut.2026.1913449)
Supplement: Supplementary file 1 [file Table_1.DOCX]

Supplementary Material

# 1 Supplementary Tables

**Table S1** Structural information for the 18 active compounds.

| Category | Compound name | CAS No. | Chemical formula | Structural formula | Food sources |
| --- | --- | --- | --- | --- | --- |
| Flavonoids | Sophorabioside | 2945-88-2 | C_27_H_30_O_14_ |  | *Sophora japonica* flower |
|  | Ginkgetin | 481-46-9 | C_32_H_22_O_10_ |  | *Ginkgo biloba* L. leaf |
|  | Dihydromyricetin | 27200-12-0 | C_15_H_12_O_8_ |  | Seed of *Hovenia dulcis* Thunb. |
|  | Kaempferol-3-O-rutinoside (Nicotiflorin) | 17650-84-9 | C_27_H_30_O_15_ |  | *Carthamus tinctorius* |
|  | Puerarin | 3681-99-0 | C_21_H_20_O_9_ |  | *Pueraria lobata* |
|  | Naringin | 10236-47-2 | C_27_H_32_O_14_ |  | *Solanum lycopersicum* L., *Citrus paradisi*, and other citrus fruits |
|  | Daidzin | 552-66-9 | C_21_H_20_O_9_ |  | *Glycine max* (L.) Merr. |
|  | Sophoricoside | 152-95-4 | C_21_H_20_O_10_ |  | *Sophora japonica* flower |
|  | Reynoutrin | 549-32-6 | C_20_H_18_O_11_ |  | *Psidium cattleyanum* Sabine |
|  | Gardenin-B | 2798-20-1 | C_19_H_18_O_7_ |  | *Gardenia jasminoides* Ellis |
|  | Rutin-trihydrate | 250249-75-3 | C_27_H_36_O_19_ |  | *Combretum micranthum* G. Don |
|  | Quercetin | 117-39-5 | C_15_H_10_O_7_ |  | *Glycyrrhiza uralensis* Fisch |
| Phenols | Salvianolic-acid B | 121521-90-2 | C_36_H_30_O_16_ |  | *Salvia miltiorrhiza* Bge. |
|  | [6]-Gingerol | 23513-14-6 | C_17_H_26_O_4_ |  | *Zingiber officinale* Roscoe |
|  | Myrislignan | 171485-39-5 | C_21_H_26_O_6_ |  | *Myristica fragrans* Houtt. |
| Others | Genipin 1-β-D-gentiobioside | 29307-60-6 | C_23_H_34_O_15_ |  | *Gardenia jasminoides* Ellis |
|  | Gamma-Linolenic-acid | 506-26-3 | C_18_H_30_O_2_ |  | *Tadehagi triquetrum* (L.) Ohashi |
|  | Ginsenoside Re | 52286-59-6 | C_48_H_82_O_18_ |  | *Panax ginseng* C. A. Meyer |
